# Factors predictive of successful retention in care among HIV-infected men in a universal test-and-treat setting in Uganda and Kenya: A mixed methods analysis

**DOI:** 10.1371/journal.pone.0210126

**Published:** 2019-01-23

**Authors:** Lillian B. Brown, Monica Getahun, James Ayieko, Dalsone Kwarisiima, Asiphas Owaraganise, Mucunguzi Atukunda, Winter Olilo, Tamara Clark, Elizabeth A. Bukusi, Craig R. Cohen, Moses R. Kamya, Maya L. Petersen, Edwin D. Charlebois, Diane V. Havlir, Carol S. Camlin

**Affiliations:** 1 Division of HIV, ID and Global Medicine, University of California San Francisco, San Francisco, California, United States of America; 2 Center for AIDS Prevention Studies, University of California San Francisco, San Francisco, California, United States of America; 3 Department of Obstetrics, Gynecology & Reproductive Sciences, University of California San Francisco, San Francisco, California, United States of America; 4 Kenya Medical Research Institute, Kisumu, Kenya; 5 Infectious Diseases Research Collaboration, Kampala, Uganda; 6 Makerere University College of Health Sciences, Kampala, Uganda; 7 Divisions of Biostatistics and Epidemiology, School of Public Health, University of California Berkeley, Berkeley, California, United States of America; Sefako Makgatho Health Sciences University, SOUTH AFRICA

## Abstract

**Background:**

Previous research indicates clinical outcomes among HIV-infected men in sub-Saharan Africa are sub-optimal. The SEARCH test and treat trial (NCT01864603) intervention included antiretroviral care delivery designed to address known barriers to HIV-care among men by decreasing clinic visit frequency and providing flexible, patient-centered care with retention support. We sought to understand facilitators and barriers to retention in care in this universal treatment setting through quantitative and qualitative data analysis.

**Methods:**

We used a convergent mixed methods study design to evaluate retention in HIV care among adults (age > = 15) during the first year of the SEARCH (NCT01864603) test and treat trial. Cox proportional hazards regression was used to evaluate predictors of retention in care. Longitudinal qualitative data from n = 190 in-depth interviews with HIV-positive individuals and health care providers were analyzed to identify facilitators and barriers to HIV care engagement.

**Results:**

There were 1,863 men and 3,820 women who linked to care following baseline testing. Retention in care was 89.7% (95% CI 87.0–91.8%) among men and 89.0% (86.8–90.9%) among women at one year. In both men and women older age was associated with higher rates of retention in care at one year. Additionally, among men higher CD4+ at ART initiation and decreased time between testing and ART initiation was associated with higher rates of retention. Maintaining physical health, a patient-centered treatment environment, supportive partnerships, few negative consequences to disclosure, and the ability to seek care in facilities outside of their community of residence were found to promote retention in care.

**Conclusions:**

Features of the ART delivery system in the SEARCH intervention and social and structural advantages emerged as facilitators to retention in HIV care among men. Messaging around the health benefits of early ART start, decreasing logistical barriers to HIV care, support of flexible treatment environments, and accelerated linkage to care, are important to men’s success in ART treatment programs. Men already benefit from increased social support following disclosure of their HIV-status. Future efforts to shift gender norms towards greater equity are a potential strategy to support high levels of engagement in care for both men and women.

## Background

The expansion of antiretroviral treatment (ART) programs in sub-Saharan Africa has dramatically increased the proportion of HIV-infected individuals accessing treatment[1]. However, men have largely been left behind from the gains of ART. Across sub-Saharan Africa, men test for HIV at lower rates[2], have higher rates of attrition from treatment programs [3–8], higher rates of virologic failure on ART [9–12], and higher mortality on ART[3, 6, 7, 13–15]. This translates into increasing gender disparity in life expectancy on ART [13, 16].

The reasons underpinning this disparity that have been explored in the literature to date have focused on gender norms and expressions of masculinities that run counter to health-seeking behaviors. Additionally, the requirements of men’s employment and livelihoods contribute to sub optimal male testing and engagement in treatment. Entrenched gender norms, promulgating the view that healthcare-seeking and healthcare spaces are primarily the woman’s domain, contributes to an avoidance of HIV testing among men[17]. After an HIV diagnosis, men can feel their masculinity is compromised by admitting that they are sick and by asking for help[18, 19]. In Tanzania, the hegemonic form of masculinity [20]—feelings of superiority in which ‘real men’ are strong, emotionally, independent, tough, and fearless, which lead men to feel ashamed at having to visit a clinic in order to access treatment—directly conflicted with the health-promoting behavior required for ART programs [21]. Studies in Tanzania, Uganda, and Kenya have found that men fear that disclosure of their HIV status may threaten their leadership positions in households and contribute to family instability[21]; they fear blame for engaging in extramarital affairs, and accusations of ‘promiscuity’ by their wives [22]. HIV is also seen as a threat to male gender role expectations related to the ‘provider’ role, as it relates to work and providing resources for the family. Among mine workers, for example, men feared that disclosure would lead to fewer job offers and work opportunities[23]. Men’s work commitments, and long clinic wait times that interfere with their livelihood activities, are reported as barriers to seeking ART care[24].

The success of antiretroviral treatment the health of the individual and for prevention hinges on achieving high levels of viral suppression through engagement in ART care. The ongoing 334,000 person Sustainable East Africa Research in Community Health (SEARCH) HIV ‘test and treat’ trial (NCT 01864603) actively sought to increase male engagement in care by addressing known barriers to care. A messaging campaign was undertaken in the communities before and during the baseline year highlighting the health benefits of ART and starting ART when asymptomatic as a way to stay healthy. This message countered the prevailing treatment paradigm in the region at the time which was to initiate ART at CD4^+^ counts less than 350 cells/mm^3^. Messaging also emphasized the importance of adherence in order to achieve the health benefits of ART. Patients found to be HIV-infected received accelerated linkage to HIV care with rapid ART start. ART was provided through a patient centered, streamlined care approach[25]. Decreased wait times[26] minimized the opportunity costs for men to be away from work, and flexible clinic hours were offered to allow men to fit clinic visits around their work day. Phone call reminders before appointments and retention tracking after missed appointments were implemented to support retention in care. Care was provided in a friendly, supportive environment and no putative measures were taken for missed visits.

In this setting which explicitly addressed known barriers to care, men were retained in HIV care at the same rate as women in SEARCH intervention communities [27]. We sought to understand what factors contributed to the high retention in care observed among men. We use a convergent mixed methods study design in which quantitative and qualitative data are collected and analyzed simultaneously[28] to explore male-specific factors related to retention in care. We describe predictors of 12-month retention in care among HIV-infected men who are linking to HIV care for the first time in rural Uganda and Kenya as part of the ongoing SEARCH universal test-and-treat trial, and explore these findings using qualitative data to gain a more complete understanding of these results, guide additional quantitative data analysis, and help interpret quantitative findings.

## Methods

### SEARCH study design

The SEARCH trial is a community cluster-randomized controlled trial in 32 communities in three regions in Kenya and Uganda, in which all communities received a community census and population-wide HIV testing at baseline. These are rural communities, defined as one or more national geopolitical units, just above the village level (i.e. a parish in Uganda and a sublocation in Kenya) with a population of about 10,000 people, within the catchment area of a government supported ART clinic. All residents in each community were enumerated in a door-to-door census. In each community, a 2-week multi-disease health campaign (CHC) was conducted that included HIV testing and counseling, and referral to HIV care if infected. Residents who did not attend the community health campaign were approached for home-based testing (HBT) within 1–6 months after the CHC. Using this hybrid mobile HIV testing approach, 89% of the population was tested at baseline [29]. In the 16 intervention communities, all HIV-infected individuals in the intervention communities were then referred for ART within a streamlined model of care, a patient-centered model designed to reduce patient-level barriers and maximize health system efficiency[25]. The central features of the streamlined care model included: (1) immediate ART initiation; (2) a patient-centered, welcoming, empathetic environment; (3) co-location of clinical, phlebotomy and medication dispensing services; (4) VL monitoring with structured VL counseling; (5) co-located care for non-communicable diseases including hypertension and diabetes; (6) quarterly (rather than monthly) clinic visits and ART dispensing; (7) 24-hour telephone access to a clinician; (8) flexible clinic hours and locations for ART dispensation; and (9) telephone appointment reminders and patient tracking following missed visits[25].

### Measures

#### Outcome

Retained in care was defined as not more than 90 days late to a 12-month follow-up visit, or documented transfer to an alternative site. Patients were considered out of care (non-retention) if they were found alive, in the community and not enrolled in HIV care, or moved out of the community without a documented transfer, or were lost to follow-up.

#### Predictors

Sex, age, education, occupation, mobility, access to a cell phone, and HIV testing location (CHC versus HBT) were obtained during the baseline year. Education was categorized as no school, any primary or completed primary school, and any secondary or further education. The 20 occupational categories at baseline were further classified as formal (student, teacher, government worker, military worker, health worker, factory worker), informal-high risk (fisherman, bar owner, bar worker, truck/taxi/motorcycle/bike/boat driver, or tourism), informal-low risk (farmer, shopkeeper, market vendor, hotel worker, household worker, construction worker), no job (unemployed, disabled), or other. Individuals were considered a stable resident if they reported having resided within the community for at least 6 months out of the 12 months prior to census enumeration and considered to be ‘mobile’ if they resided within the community for less than six of the past twelve months.

### Statistical analysis

The analysis of predictors of retention was restricted to adult (≥ 15 years) men who did not have a previous record of HIV care and had at least one clinic visit after baseline hybrid testing and before follow-up year 1 testing or September 16, 2014, in order to have opportunity for 12 months of follow-up at the time the data were queried (December 16, 2015). Patients entered the risk group (T_0_) at their first clinic visit after baseline hybrid testing. Time to attrition was calculated as the time between T_0_ and their last scheduled clinic visit. Kaplan Meier estimates of survival in which patients were censored at the time of transfer or death were used to calculate probability of retention at one year. Sub-hazard ratios for attrition were computed using proportional hazards modeling with death as a competing risk. In the competing risks analysis patients were censored at the date of transfer and all data were truncated at 365 days of follow-up. The proportional hazards assumption was assessed graphically and with Schoenfeld residuals. The multivariate model included region, sex, and age based on *a priori* determination, and covariates that were significant in univariate analysis were added in stepwise progression and included in the model if they contributed significantly to the fit of the model using likelihood ratio test with p<0.1. Robust standard errors were used and community was included as a fixed effect in all models to control for clustering by community. Stata v14 (College Station, Texas) was used for analysis.

### Qualitative procedures

An ongoing qualitative study is embedded within the SEARCH trial, and includes data collection in random and purposively selected cohorts of adult participants in 8 of the 32 SEARCH communities, to characterize diverse social and cultural contexts, implementation factors, and social and behavioral mechanisms of intervention action, across the settings for the trial. Among the topics the qualitative study is exploring are barriers to and facilitators of engagement and retention in HIV care and treatment programs.

#### Participants and procedures

This study draws on longitudinal qualitative data collected via in-depth interviews conducted in study communities during the first and second years of SEARCH (February 2015- January 2016). Data were composed of transcripts of thirty-two interviews conducted with community leaders (n = 16 at baseline and follow up), purposively selected based on their role in the community and SEARCH in order to probe around the norms around access to care in their community; forty-nine interviews with providers (n = 28 at baseline and 21 at follow up, including n = 6 replacements), purposively selected to reflect the variety of roles in HIV care, and one hundred nine interviews with randomly selected HIV-positive community members, across four intervention communities (n = 56 at baseline; n = 53 at follow up due to deaths and refusals). Data on HIV status and age were not collected for participating community leaders and providers.

#### Qualitative data collection and analysis

The lead investigator for the qualitative study in SEARCH trained a team of local researchers in qualitative data collection, coding and analysis. The local researchers used in-depth semi-structured guides to conduct interviews in local languages Ateso, Dholuo, Lusoga, Lugwere, and Runyankole. Guides for community member and leader interviews explored barriers to and facilitators of HIV care, while provider interviews explored individual and community-level challenges to care engagement and ART adherence S1 File. In addition, all interviews explored cross-cutting topics such as individual and community attitudes towards HIV/AIDS, and perceptions and experiences related to stigma and disclosure. Study team members conducted interviews, transcribed audio recordings and translated into English. Drawing upon constructivist grounded theoretical approaches[30], a master code list was developed and defined by the lead investigator, study coordinator and local researchers on the basis of theory and review of the initial empirical data, with these *a priori* codes then and applied by the eight person team. These codes were iteratively refined during the data collection and analysis process, following periodic reviews of data and group discussions to achieve consensus on applications of codes to transcript segments. Data analyses were conducted by the full study team using *Atlas*.*ti 7*.*0*.

#### Mixed methods analysis

A convergent mixed methods approach was used, in which quantitative and qualitative data were collected simultaneously and merged during the analysis phase [28].

### Ethics

The Makerere University School of Medicine Research and Ethics Committee (Uganda), the Ugandan National Council on Science and Technology (Uganda), the Kenya Medical Research Institute Ethical Review Committee (Kenya), and the University of California San Francisco Committee on Human Research (USA) approved the study protocol including the consent procedures. All participants provided verbal informed consent in their preferred language with fingerprint biometric confirmation of agreement at CHCs. Participants provided written consent in their preferred language prior to in-depth interviews.

## Results

### Demographics and overall retention in care

Among the 5,683 adult residents living with HIV who linked to HIV care following the baseline CHC, 1,863 (32.8%) were men. Men were more likely than women to be older (85.9% age ≥ 30 years vs 67.9%, *p*<0.001), have achieved a higher level of education (19.3% with any secondary or further education vs 9.4%, *p*<0.001), and be mobile (i.e., away from residence for more than half of the past year)(3.5% vs 1.9%, *p*<0.001). Men were also less likely than women to have tested at the CHC (70.5% vs 80.7%, *p*<0.001), to have previously linked to HIV care (65.5% vs 74.9%, *p*<0.001), or to be on ART before the baseline CHC (58.1% vs 63.0%, *p*<0.001) Table 1.

**Table 1:** Demographic and clinical characteristics of adult (age > = 15 years) men and women who accessed HIV care following baseline CHC.

	Men (N = 1863)	Women (N = 3820)	*p*
**Region**			
Uganda-West [n (%)]	481 (25.8%)	825 (21.5%)	0.002
Uganda-East [n (%)]	219 (11.8%)	455 (11.9%)	
Kenya [n(%)]	1163 (62.4.4%)	2540 (66.5%)	
**Age Group**			<0.001
15–19 years	15 (0.8%)	122 (3.2%)	
20–24 years	64 (13.7%)	402 (10.5%)	
25–29 years	183 (9.8%)	708 (18.5%)	
30–34 years	287 (15.4%)	640 (16.8%)	
35–39 years	344 (18.5%)	598 (15.7%)	
40–44 years	282 (15.1%)	477 (12.5%)	
> 45 years	688 (36.9%)	873 (22.9%)	
**Education**			<0.001
No School	130 (7.0%)	407 (10.7%)	
Primary or less	1369 (73.5%)	3044 (79.7%)	
Any Secondary or further	359 (19.3%)	360 (9.4%)	
Don’t know/refused to answer	5 (0.3%)	8 (0.2%)	
**Occupation**			<0.001
Farming	1109 (59.5%)	2352 (61.6%)	
Fishing	203 (10.9%)	218 (5.7%)	
Shopkeeper/Vendor	86 (4.6%)	472 (12.4%)	
Household worker	17 (0.9%)	238 (6.2%)	
Transport worker	70 (3.8%)	2 (0.1%)	
Student	13 (0.7%)	59 (1.5%)	
Other	313 (16.8%)	258 (6.8%)	
No Job	52 (2.8%)	221 (5.8%)	
**Mobility status**			<0.001
Stable	1798 (96.5%)	3746 (98.1%)	
Mobile	65 (3.5%)	74 (1.9%)	
**Access to cell phone**	1254 (67.3%)	2467 (64.6%)	0.05
**CD4 at baseline CHC**			
<50 cells/mm^3^	19 (1.0%)	23 (0.6%)	<0.001
50–200 cells/mm^3^	179 (9.6%)	161 (4.2%)	
200–350 cells/mm^3^	399 (21.4%)	493 (12.9%)	
350–500 cells/mm^3^	450 (24.2%)	825 (21.6%)	
>500 cells/mm^3^	633 (34.0%)	2063 (54.0%)	
Missing baseline CD4	183 (9.8%)	255 (6.7%)	
**Pre-ART CD4 above country treatment guidelines**	417 (22.4%)	840 (22.0%)	0.7
**HIV RNA < 500 copies/ml**	679 (36.4%)	1598 (41.8%)	<0.001
Missing baseline viral load	498	949	
**HIV Testing Location**			
CHC	1312 (70.5%)	3070 (80.7%)	<0.001
Post-CHC tracking	550 (29.5%)	736 (19.3%)	
**Previous linkage to care**			
Yes	1221 (65.5%)	2861 (74.9%)	<0.001
No			
**On ART before baseline CHC**	1075 (58.1%)	2383 (63.0%)	<0.001
**Time to link to HIV care among newly linking**			
< = 30 days	952 (51.1%)	1786 (46.8%)	0.002
>30 days	911 (48.9%)	2034 (53.3%)	

**Fig 1:**
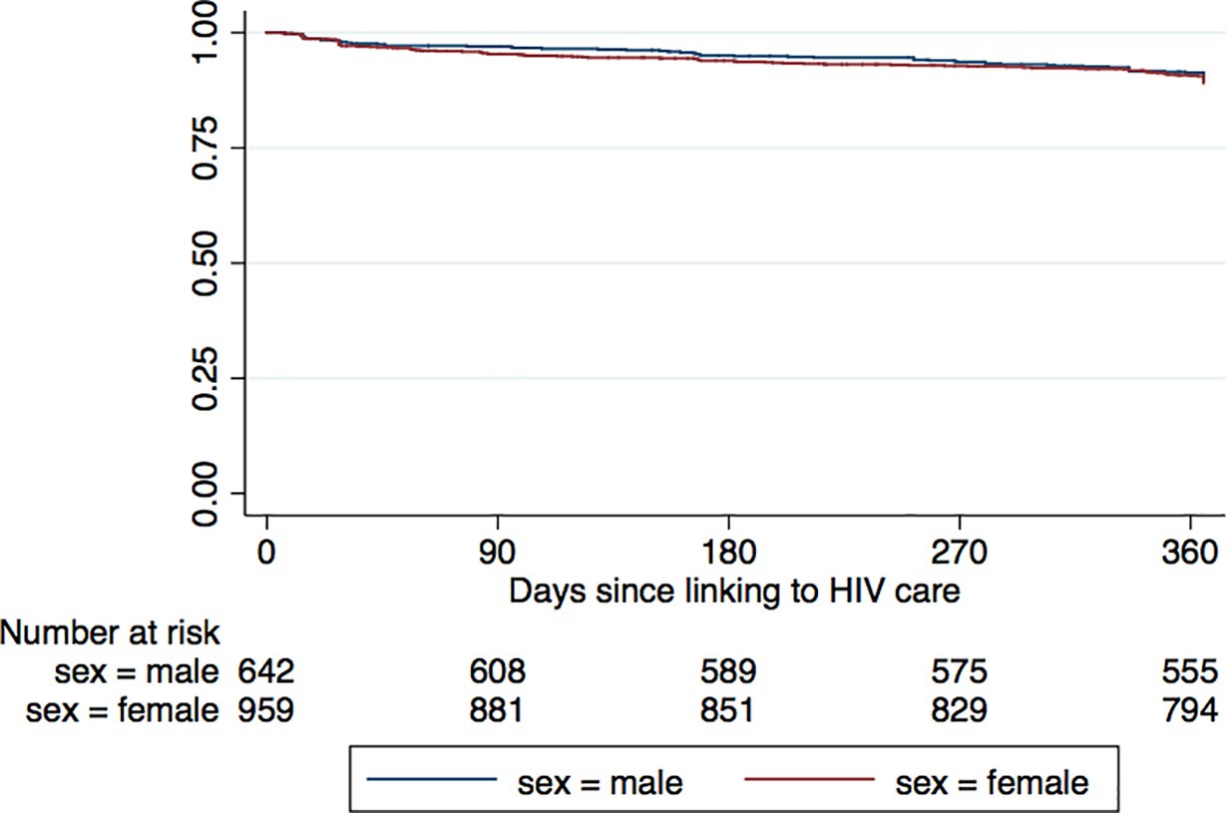
Retention in HIV care over one year by sex.

Among men, the probability of retention at one year was 89.7% (95% CI 87.0–91.8%). Among women, the probability of retention at one year among was 89.0% (95% CI 86.8–90.9%)(Fig 1). Among those retained in care at one year, viral suppression was similar among men and women (86.7% in both, *p* = 0.9).

### Predictors of retention

Retention was not associated with region, education level, occupation, access to a cell phone, or mobility. Younger age was a significant predictor of attrition at one year among both men and women, while initiating ART with CD4+ count above country treatment guidelines was associated with higher retention at one year among men Table 2. Men who linked to care and initiated ART quickly were more likely to be retained in care at one year, as taking more than 30 days to link to care after the time of testing was associated with lower retention (aHR for non-retention 1.67, 95% CI 1.02–2.74).

**Table 2:** Predictors of not being retained in care at 12 months in adult residents of SEARCH intervention communities newly linking to HIV care after baseline hybrid mobile testing among men (N = 642) and women (N = 959).

	Men (N = 642)				Women (N = 959)			
Predictor	Hazard Ratio	95% CI	Adj Hazard Ratio	95% CI	Hazard Ratio	95% CI	Adj Hazard Ratio	95% CI
**Region**								
Kenya	ref		ref		ref		ref	
Uganda-East	1.49	0.88–2.54	1.17	0.56–2.45	1.45	0.92–2.30	1.85	1.07–3.22
Uganda-West	1.37	0.66–2.86	1.13	0.61–2.09	1.41	1.01–1.96	1.54	0.96–2.47
**Age**								
15–24	4.13	2.69–6.35	4.07	2.03–8.15	3.83	2.45–5.96	3.68	2.31–5.86
25–29	2.59	1.62–4.13	2.80	1.57–4.99	2.13	1.26–3.58	2.24	1.32–3.80
> = 30	ref		ref		ref			
**Education**								
No School	0.88	0.41–1.87			0.54	0.26–1.11		
Primary	ref				ref			
Any secondary or further	0.85	0.50–1.45			0.99	0.52–1.88		
**Occupation**								
Formal	ref				ref			
Informal–high risk	1.31	0.40–4.25			1.14	0.06–21.7		
Informal–low risk	1.89	0.29–7.02			2.86	0.41–20.1		
No job	1.89	0.29–12.3			6.74	0.8–55.9		
Other	2.01	0.34–11.8			2.92	1.24–6.90		
**Own a cell phone**								
Yes	ref				ref			
No	1.49	0.79–2.81			1.21	0.84–1.75		
**Mobility**								
Stable resident	ref				ref			
Not stable resident	2.26	0.69–7.45			1.30	0.26–6.43		
**Pre-ART CD4**								
Below country treatment guidelines	ref		ref		ref		ref	
Above country treatment guidelines	0.71	0.44–1.16	0.56	0.33–0.94	0.63	0.43–0.93	0.65	0.44–0.96
**Site of testing**								
CHC	ref		ref		ref		ref	
Post-CHC tracking	0.51	0.29–0.88	0.54	0.26–1.12	1.24	0.76–2.0	1.35	0.84–2.17
**Time to link**								
< = 30 days	ref		Ref		ref		ref	
> 30 days	1.78	1.05–3.01	1.67	1.02–2.74	1.36	0.95–1.97	1.32	0.89–1.97

### Qualitative findings

Integrating qualitative data from this study population provides insights into the dynamics underlying men’s high retention in ART programs in SEARCH intervention communities relative to prior research among men in the region. In the results that follow we present qualitative findings that help contextualize these clinical outcomes and, when available, key analyses of the clinical data on retention that complement these findings.

*Physical health was a strong motivation for ongoing ART care engagement*. Men who initiated ART above the country treatment guidelines at the time (350 cells/mm^3^) were less likely to fall out of care than those men initiating ART at lower CD4 counts [aHR 0.56 (95% CI 0.33–0.94)]. In addition, clinical data show that men initiating ART at lower CD4 counts (350 cells/mm^3^) who would be expected to feel tangible health benefits from ART, were more likely to be retained in care at one year compared to women who initiated care at low CD4 counts (81% vs 74%, *p* = 0.03). HIV-positive men credited ART for improving their health status, increasing their ability to engage in work and to appear physically healthy. Some newly diagnosed men with higher CD4 counts were motivated to start ART in order to stay healthy and not spread the virus to others:

*“After learning about my status*, *they told me that there are drugs*, *and these drugs are for free*. *I was also told that the drugs they give me will help to reduce the amount of viruses in my blood and weaken it*. *The virus will not spread*. *I will not get coughs daily*. *I felt happy and decided to continue […]*.*” (Male*, *30*, *HIV-positive*, *Uganda)*

Men initiating ART at lower CD4 counts specifically discussed the immediate changes observed following ART initiation as a motivation for remaining in care. This improvement in health and physical ability was also viewed as a positive change that shielded men from the potential stigma associated with the physical signs of an HIV-infection.

*“Before I started using them [ART medications]*, *I could not perform my tasks normally even with minor ailments*. *I think only an accident can destabilize my capability but not things like malaria […] it [ART] is part of me and when I miss it*, *I even feel like my mind is not working properly*.*”* (Male, 41, HIV-positive, Kenya)

In addition to improved health following ART initiation, men also internalized messaging on treatment adherence, especially the importance of taking ART consistently.

*“We were taught that if we miss [ART]*, *one stands being resistant to drugs*. *I look at my status and my age*, *and then I say… I must push on to at least see my children grow to be old people*.*”* (Male, 30, HIV- positive, Kenya)

*“I do not skip [my medication]*. *My phone reminds me of the time*. *Even though I go for a visit I do not forget my drugs*. *In case I do not expect to be home soon*, *I simply put my drugs in a small envelop and move with it such that when the time comes I simply swallow it*.*”* (Male, 38, HIV- positive, Uganda)

*“If I have a commitment on the appointment date*, *[…] I would rather collect the drugs earlier than the appointment date in order not to default*.*”* (Male, 42, HIV-positive, Kenya)

“Feedback” via viral load testing further reinforced men’s motivation to adhere to medications:

*“…After three months*, *I went to the health facility and I was told that the virus in my blood was no longer detectable and my blood was good*. [Interviewer: How did you feel when the provider told you that?] *I was so excited*. *[…] I saw the viral load testing as something very important*. *I learnt that it was important for me to stick to my medications and not to give up on them*.*”* (Male, 30, HIV-positive, Uganda)

*“Of late I was told it* [viral load] *is down […] so I felt happy at the good news*. *My mathematics is that I add more years when it is low*.*”* (Male, 37, HIV-positive, Kenya)

*The patient centered approach to care with accelerated linkage to care and enhanced retention support through reminder phone calls and retention tracking also facilitated men’s adherence*. Men who linked to care and started ART soon after testing positive (within 30 days) were more likely to be retained in care. In addition, we found that among men retained in care, 17% required retention tracking through phone calls or in person visits and flexible treatment options to support their engagement in care. Men especially appreciated the improved speed with which they were seen at facilities:

*“When we go to the health facility […] the providers attend to us very fast and they treat us humanely*.*”* (Male, 30, HIV-positive, Uganda)

*Supportive partners and the ability to disclose their HIV-status without penalty helped men engage in care*. Among serodiscordant couples, HIV-negative women were supportive of their male partner’s engagement in care, often encouraging treatment adherence and reminding their husbands about clinic appointments.

*“I tested at a mobile VCT center and was referred […] this is when my wife knew that I had tested before and had not honored the initial referral*. *I disclosed to her that I wanted to confirm my result first before starting on care*. *She encouraged me to go to the hospital*. *At the hospital*, *I got tested for the third time and this time around got enrolled in HIV care*.*”* (Male, 49, HIV-positive Kenya)

Consistent with these observations, HIV-positive men in a serodiscordant couple were more likely than HIV-positive women with a negative partner to be retained in care at one year. Among married couples, the proportion of men and women in seroconcordant relationships retained at one year was similar (87% vs 85%, *p* = 0.4); however, men in discordant relationships were significantly more likely than women with a discordant spouse to be retained at one year (88% vs 80%, *p* = 0.02).

*Men experienced fewer negative consequences than did women in response to an HIV-positive status disclosure*. Interview narratives also showed far less severe outcomes in response to an HIV-positive status disclosure among men compared to women. While data show that both men and women experience challenges with HIV-status disclosure, men’s HIV-positive status disclosure often ended on auspicious terms, with their female partners remaining in the relationship and supporting them. In this excerpt, a man living with HIV described his HIV-status disclosure to his wife, and her eventual acceptance:

*“One morning I asked her to accompany me to the hospital and I did not even disclose to her that I had earlier tested positive and had decided to start care on that very date […] I only realized that she knew my status from other people whom she had talked to about my poor health*. *She later came to terms with my status and I confirmed to her that I was HIV positive*.*”* (Male, 38, HIV-positive, Kenya)

Some HIV-positive men described their partner’s immediate acceptance of their HIV-status upon disclosure:

*“I was so happy when they tested us*, *I knew my results but for her she was negative*. *But we continued living in peace and she didn’t decide to leave me*, *because she felt that I was open to her*.*”* (Male, 32, HIV-positive, Uganda)

*Non-disclosure had more negative consequences for women compared to men*. Women had difficulty keeping HIV care appointments or adhering to medication when concealing their HIV-positive status. Women’s narratives about avoiding disclosure included recollections of having hidden drugs, while none of the HIV-positive men described hiding drugs for fear of negative consequences. Providers also described instances of female HIV-positive patients hiding drugs to avoid disclosure and for fear of a negative reaction from their male partners:

*“We have a challenge in couples where if a pregnant woman tests positive for HIV and we give her drugs*, *she is scared to tell her husband that she is positive; she fears to take the drugs and hides them away from her husband and keeps them at the neighbor’s place*. *Sometimes*, *she forgets to take the drugs and when we talk to such women*, *they tell us that they forgot to take the drugs because they hide them outside their own homes*.*”* (Provider, Uganda)

Similarly, a provider recounted the story of a female HIV-positive patient’s complex care navigation and default of medication due to non-disclosure to her male partner:

*“She then lost the confidence to tell him about her HIV status […] she even misses coming for her drugs at times […] She comes at different dates from that assigned to her so that people don’t see her*, *because she is scared to be seen by her husband as he will think that she is the one that infected him with HIV”* (Provider, Uganda)

*Men who do not disclose their HIV status are able to seek care in remote facilities*. Interview narratives suggested that non-disclosure was less likely to result in men not being in care at all, as compared to women, as men had more agency and decision-making power to seek care without their partner’s knowledge. Providers, partners of HIV-positive men, and HIV-positive men, all reveal that men often engaged in care in remote facilities, without disclosing their HIV-positive status.

*“You find that some women discover when it is too late that their husbands are living positively and have already started taking their drugs*, *because they* [husbands], *too*, *fear disclosing their status to their wives*.*”* (Provider, Uganda)

“*She used me to disclose to her husband*, *but I discovered that her husband had started HIV care long ago and was not able to disclose to the wife*.*”* (Female, 36, HIV-positive, Kenya)

*“He started on drugs without my knowledge […] I felt bad but what to do*? *I had to calm down […] He first kept quiet for about five months*, *and I too kept quiet*, *until one day I saw him with ART…”* (Female, 34, HIV-positive, Uganda)

*“There are men that may leave this place and go to test and when they find that they have HIV*, *they refuse to tell their wives and continue taking their medications in secret*. *The man looks strong and the woman is weak and you ask yourself why that is like that*?*”* (Female, HIV-unknown, Kenya)

Polygamous men, in particular, were able to remain in care while selectively disclosing their HIV-positive status; men hid their medication in the household of the partner to whom they had disclosed their HIV-positive status.

*“I also realized that he and my co-wife were already in care*. *He first pretended to be innocent and urged me not to believe the result*, *and instead test more times to confirm”* (Female, 36, HIV-positive, Kenya)

*“*[I: Which gender is mostly doing this?] *Mostly they are men*, *or sometimes men with their younger wives* [I: So the other wife is in the dark?] *Completely*, *she does not know that the two are on drugs”* (Female, 44, HIV-positive, Kenya)

*“[…] to make matters worse*, *both the younger wife and the husband had all enrolled for HIV care without telling the elder wife”* (Female, 36, HIV-positive, Kenya)

## Discussion

In contrast to a large literature from sub-Saharan Africa in the pre-universal test-and-treat era showing that men have had worse outcomes on antiretroviral therapy compared to women, men in this universal test-and-treat study setting, in which some of the known barriers to engagement in care had been addressed by intervention strategies, had similar high rates of retention in care and, importantly, had similar rates of viral suppression at one year. Aspects of the SEARCH intervention, such as positive messaging about ART effects on health, and interventions implemented to make care access easier for men, likely contributed to the high retention rates seen among men: these intervention elements included facilitated linkage and rapid ART start, ART initiation at higher CD4 counts, flexible clinic hours, and retention tracking for missed appointments. We also found that structural support and social empowerment experienced by men in the study settings also facilitated their engagement in care. Not only were men able to seek care in remote clinics and hide their ARVs as needed, they also received partner support to remain in care when they did disclose; women’s experiences with disclosure were far more mixed, as we have documented in prior research[22].

Despite other studies suggesting that “feeling healthy” may be a reason men are lost-to-follow-up [17, 31, 32], in this setting maintaining improved health was a motivating factor to remain engaged in care. Men described feeling committed to care because of their experiences of the positive benefits of adhering to ART. In fact, we found that men who initiated ART with high CD4+ counts above country treatment guidelines (and were likely to be asymptomatic) were more likely to be retained in care and virally suppressed than men with lower CD4+ counts, which may reflect the desire to maintain health and the masculine ideal of strength. Improved health as a motivator to stay in care may not only relate to a new sense of self-efficacy related to care-seeking, but also gains saliency as it upholds a normative masculinity in which providing for one’s family is valorized. Feeling healthy may improve employment prospects, and reduce the need for frequent clinic visits, which previously may have disrupted employment, prior to the implementation of streamlined care within the SEARCH intervention. Messaging that ART can allow men to maintain their health, avoid compromising their masculinity, and provide for their families can be powerful motivators for engagement in care.

The streamlined care model addresses some of the structural and logistical barriers to care men face. In particular, rapid ART start, decreased frequency of scheduled visits, decreased wait times, and flexible clinic hours minimize conflicts with work. However, despite these improvements, nearly one in five men in our setting benefited from retention tracking, in which a personal contact was made by a health care worker to men who had missed appointments and arranged a convenient time for them to come to clinic or provide off-site services, including medications. Community-based ART delivery and mobile ART pick-up sites may minimize these barriers even further and may reduce the reliance on retention tracking. Notwithstanding these improvements, in this region care-seeking behavior has long been less normative for men[17, 18, 33], which likely reflects both social norms related to masculinities [21, 23, 34] and the history of institutional policies that prioritized testing and access to treatment for women through the focus on testing and treatment in antenatal clinics[35]. In addition to removing logistical barriers, approaches to make the clinic more inviting to men may help combat the perception of the clinic for women and children and integrating additional services, such as chronic disease care, may also help normalize health care for men.

Consistent with previous literature, narratives from qualitative interviews in this study support observations that men are more socially empowered than women, and this facilitates their engagement in care[22]. While the SEARCH study had to undertake specific activities to encourage men to participate in testing, and men who tested positive often delayed linkage, when men did decide to seek care, they faced fewer and less severely negative consequences of disclosure (despite their fears of diminished sexual opportunities and social status in their homes or communities), and fewer financial and structural barriers compared to women. In other settings in east Africa men were more likely to access ART informally by taking their wives’ drugs or buying from other patients[21], and were more likely to enroll in care without telling their partners[22]. We found that because of fewer constraints on their mobility, men were more readily able to seek care in remote facilities and to take ART in secret than were women. In addition to these advantages, men were more supported by their partners when seeking care than were women. This is consistent with previous observations underscoring the importance of family support, including intimate partner support, for facilitating care retention [32]. Thus, despite men’s well-documented health disadvantages due to male gender norms that discourage their health-seeking behavior, other aspects of their structural advantage in gendered power relations were found to also facilitate their retention in HIV care.

This analysis is subject to several limitations. The high retention reported among men is conditional on both testing and linking to ART care so may select for those most men most likely to remain engaged in care. Testing coverage was 86% among all men in this population[29] and future analyses will examine differences between men who remain untested and men who do not link to care. Only retention in the first year is captured so longer follow-up will be necessary to evaluate durability of retention in care. In addition, self-reported data from individuals and providers are subjective and potentially affected by recall and social desirability biases. Qualitative analyses are strengthened by the volume of interview data collected from heterogeneous sources and community settings, with strong convergence of themes emergent in the data across these sources and over time.

## Conclusion

Improving male engagement in care is essential both for their individual health and for treatment as prevention to succeed at a population level. Through an intervention addressing traditional barriers to care and the underlying society structure that supports men, men in this universal care setting experienced high retention. However, retention in care and viral suppression among both genders still falls short of ideal, particularly those who are linking to care for the first time and are at highest risk of attrition and among youth of both genders. Additionally, women especially continue to need support from care providers to facilitate disclosure to partners, partner testing, and partner support. Efforts to improve gender equity in communities and society continue to be needed to support both women’s and men’s engagement in and sustained retention in care and treatment.

## Supporting information

S1 File Combined In-depth interview guides. (PDF)
