# Supplementary material for: Factors predictive of successful retention in care among HIV-infected men in a universal test-and-treat setting in Uganda and Kenya: A mixed methods analysis
Source: PLoS One. 2019 Jan 23;14(1):e0210126. doi: 10.1371/journal.pone.0210126 (PMC6343966; doi:10.1371/journal.pone.0210126)
Supplement: S1 File — (PDF) [file pone.0210126.s001.pdf]

SEARCH Trial Qualitative Cohort- BASELINE INTERVIEW

Thank you for taking the time to talk with me today. I would like to ask you some questions about your experiences as a member of this community. I am interested in hearing about your experiences, opinions and beliefs in your own words. If there are any questions that make you feel uncomfortable, you should feel free to tell me and we can skip over those questions.

Can you tell me about this community [*insert local place name*]?

Probes: How long have you lived here? Were you born here? Were you married into this community? Did you migrate here from another community? Do you consider this community to be your home? How would you describe this community? If you could change anything about this community what would it be?

What are some of the major problems that are faced by members of this community?

Probes: How long have these problems existed? Have these always been a big problem, or did these problems come up recently? How do people in this community deal with these problems? What are some of the health problems faced by this community? If the participants do not mention HIV/AIDS as one of the major problems, probe specifically about this. Is HIV/AIDS a problem in this community? How are people in this community coping with the problem of HIV?

How do people feel about HIV/AIDS in this community?

Probes: Do you know people in your community who are infected with HIV or who have died of AIDS? How do you feel about these people? How are these people treated by others in this community? If someone you know told you that they were infected with HIV, how would you react? Why do some people fear to disclose their HIV status to their partner, family members, or others in the community? Do you talk to people about HIV/AIDS regularly? Can you tell me about the last conversation you had with someone when you talked about HIV? What specifically did you talk about? Why were you talking about this at that time?

Antiretrovirals, called [site-specific term], are medications used to treat HIV/AIDS. They are medicines that are used to suppress or stop HIV, the virus that causes AIDS. Are you aware of drugs called antiretrovirals that are available to help treat people with HIV?

Probes: If yes, tell me what you know about these drugs. How did you learn about these drugs? Do you know anyone in this community who is using these drugs? Where do they get the drugs that they need? Do people have to pay money to get these drugs? Do you have any concerns about drugs that are available to treat HIV? If yes, what are your concerns? How does the availability of antiretrovirals affect the way people in your community react to HIV infected people? How do people here view other people who are taking antiretrovirals?

How would someone in this community learn their HIV status if they wanted to know?

Probes: Do you ever talk to anyone about tests that can be done to learn your HIV status? Where do most people in this community go to take the test? Why do you think people would want to get their blood tested for HIV? Do you think that the availability of drugs called antiretrovirals to treat HIV might influence whether a person is willing to test for HIV? Why do

you think people might not want to get their blood tested for HIV? If this test were available in your community, would you be interested in taking this test? Have you ever tested for HIV?
<u>If participant responds that he or she has previously tested for HIV:</u>

Tell me about your HIV testing experience.
Probes: When did you test? Why did you test? Where did you test? How did you feel about your testing experience? Did you talk to anyone about your decision to test before you got tested? Have you told others that you have tested for HIV? If yes, whom have you told? If not, why not? How did people react when you told them you tested?

Can you tell me about your experiences sharing your HIV test results with other people after you were tested?
Probes: Have you shared your HIV test results with anyone? If yes, whom? Did you share with your husband/wife/partner? Other family members? If yes, tell me about this experience – tell me about your decision to share your test results; tell me about their reaction when you shared your test results. Is there anyone you want to know your results that you have not told yet? If you did not share your results with anyone, why not? How has the availability of drugs called antiretrovirals to treat HIV affected your decision about sharing test results with people? Is there anyone who you do not want to know your HIV test results?

<u>If participant discloses that he/she is HIV seropositive:</u>

Probes: Can you please tell me, have you ever disclosed your HIV-positive status to anyone? If yes, who was the first person you disclosed to? Why did you choose that person? Were there others you wanted to disclose your status to, but didn't feel you could? Can you tell me more about that? If no, can you tell me more about why you haven't felt able to disclose your status to anyone?

Have you ever received any services and/or treatment for HIV disease?

<u>If the participant responds that he or she has NOT previously received care for HIV:</u>

Can you tell me the main reasons why you have not yet received HIV care?
Probes: Are there any other problems or issues that have kept you from getting services at the clinic for HIV? Please tell me, what would make it easier for you to enroll in HIV care and treatment?

<u>If the participant responds that he or she has previously received care for HIV:</u>

Are you currently receiving regular HIV care?
Probes: How has receiving HIV care/treatment affected your life? Where do you go for medical care? Probe for all locations. Why did you decide to seek care at that site? Have you ever had to change where you access HIV care at any point since you tested positive? If yes, probe for main reasons why. If not currently enrolled in care, can you tell me the main reasons you're not receiving care currently? Probe for ALL reasons, including mobility, negative

provider experiences, switched to traditional medicine, etc. Please tell me, what would make it easier for you to enroll again in HIV care and treatment?

Have you ever missed HIV care appointments, or dropped out of care for a time?

Probes: What were the circumstances that led this to happen? For about how long was your care interrupted? What are the main barriers you face, to being able to make appointments? Probe for ALL reasons for missing appts.: negative experiences with providers, issues of stigma and disclosure, problems with money or transportation, depression, other health issues, lack of someone else to take care of children, etc. Are there any other reasons? Please tell me, what would make it easier for you to make appointments?

What are the main barriers you face to being able to adhere to taking your HIV medications?

Probes: Probe for ALL reasons for missing medication doses.: negative experiences with providers, issues of stigma and disclosure, problems with money or transportation, need to hide medication-taking from others, depression, forgetfulness, denial, etc. Are there any other reasons? Please tell me, what would make it easier for you to adhere to taking your HIV medications?

<u>If the participant responds that he or she has NOT previously tested for HIV:</u>

You mentioned to me that you have never been tested for HIV before. Can you tell me more about this.

Probes: How do you feel about testing for HIV? What are some reasons you have not been tested for HIV before? Do you think you would ever want to be tested? If testing were available in your community, would you be interested in taking this test? Is there anything you think might change your mind about testing?

Ask all participants, whether they have tested for HIV or not.
Now I would like to ask you some questions about your sexual relationships. If you are uncomfortable with any of these questions, you can skip those questions or stop the interview completely.

Can you tell me about the partner you consider to be your primary relationship partner (defined as a partner they have been with for at least 3-months and to whom they feel a special obligation or emotional attachment)?

Probes: How did you meet this partner? How long have you been with this partner? How often do you see this partner? Do you and your partner ever talk about HIV and your risk of HIV? If you do talk to your partner about HIV and HIV risk, can you tell me what you talk about? Do you feel that you are at risk for HIV because of this partner? If yes, tell me why you think you may be at risk. Please tell me about anything you are doing in your relationship to try to minimize this risk, or if you don't feel able to do anything, please tell me about that. How has the availability of drugs called antiretrovirals to treat HIV affected this? Is there anything else you would like to do in your relationship to minimize your risk, yet you feel you are unable to do? If so, can you tell me why you feel like you are unable to do this with your primary partner?

Can you tell me about your other current sexual relationships?

Probes: How many other sexual partners do you currently have (in addition to your primary partner)? How long have you been with this/these partner(s)? Before this/these partner(s) when was the last partner you had? [If participant mentions more than one current partner], Are your partners aware that you have other sexual partners?

SEARCH Trial In-depth Interview Guide – Community Leader Cohort
Baseline Interview

Topic 1: Processes involved in mobilization of communities to participate in CHCs

Thank you for taking the time to talk with me today. I would like to ask you some questions about your experiences as a leader of this community. I'd like to start by talking with you about your involvement in the SEARCH study.

When did you first hear about the study? Thinking back to that time, what were your first thoughts about the study? What concerns did you have?

As a community leader, what has motivated you to promote the health fair?

Probes: Are there any other reasons for your involvement in the SEARCH study? How does your involvement in the SEARCH study benefit you, personally? What do you see to be the drawbacks, if any, for you personally, to being involved in the SEARCH study? What are the main benefits for the community, in your opinion?

Now let's talk about all of the things that were done in this community to prepare for the health fair. Please tell me about all of the activities that you and other took in preparation for the fair.

Probes: What do you think were the main ways that people in the community heard about the fair? Were there any other actions that you or others undertook to get people in the community to come to the fair?

Let's talk about the health fair itself. Please tell me what aspects of the fair went well, in your opinion? Which aspects of the fair did not go as well as you'd hoped?

Probes: In your opinion, what were the main reasons that community members participated in the health fair? For those that did not attend, what do you think were the main reasons they did not? Which groups of people in this community were more difficult to mobilize to attend the health fair, and why? How successful were you in getting these groups of people to attend the fair? How do you think they can better be motivated to participate in next year's fair?

Please tell me your thoughts about next year's health fair.

Probes: How do you think the fair should be promoted, next year? What will you repeat, and what things will you do differently to promote the fair among community members? For next year's fair, what aspects of the fair should be the same? How can the health fair be improved?

Topic 2: Attitudes, beliefs and social norms surrounding HIV, testing, disclosure, and ART

Now I would like to talk with you about health in this community, including the knowledge and beliefs people in this community have about HIV/AIDS, as well as your own experiences and opinions. If there are any questions that make you feel uncomfortable, you should feel free to tell me and we can skip over those questions.

What are some of the major problems that are faced by members of this community?

Probes: How long have these problems existed? Have these always been a big problem, or did these problems come up recently? How do people in this community deal with these problems? If the participants do not mention HIV/AIDS as one of the major problems, probe specifically about this: Is HIV/AIDS a problem in this community? How are people in this community coping with the problem of HIV?

How do people feel about HIV/AIDS in this community?

Probes: Do you know people in your community who are infected with HIV? How do you feel about these people? How are these people treated by others in this community? If someone you know told you that they were infected with HIV, how would you react to them? Why do some people fear to disclose their HIV status to their partner or family members? Do you talk to people about HIV/AIDS regularly? Can you tell me about the last conversation you had with someone when you talked about HIV? What specifically did you talk about? Why were you talking about this at that time?

Antiretrovirals, called [site-specific term], are used to treat HIV/AIDS. They are medicines that are used to suppress or stop HIV, the virus that causes AIDS. Are you aware of drugs called antiretrovirals that are available to help treat people with HIV?

Probes: If yes, tell me what you know about these drugs. How did you learn about these drugs? Do you know anyone in this community who is using these drugs? Where do they get the drugs that they need? What concerns do you have about access to these drugs for people in this community? Do you have any concerns about the drugs that are available to treat HIV? If yes, what are your concerns? How does the availability of antiretrovirals affect the way people in your community react to HIV infected people? Can you tell me, what are all the benefits of antiretroviral therapy? If the participants do not mention ART as prevention, probe specifically about this: Are there any other benefits? Are you aware that staying on ART can help HIV-positive people to not transmit the virus to others? To what extent do you think other people in this community know about this benefit of ART?

How would someone in this community learn their HIV status if they wanted to know?

Probes: Do you ever talk to anyone about tests that can be done to learn your HIV status? Where do most people in this community go to take the test? Why do you think people would want to get their blood tested for HIV? Do you think that the availability of drugs called antiretrovirals to treat HIV might influence whether a person is willing to test for HIV? Why do you think people might not want to get their blood tested for HIV? For women in this community, is it acceptable for them to decide to test on their own? What about men? To what extent has this changed over time? How do most people in this community feel about getting tested for HIV? And have you ever tested for HIV?

Now I want to ask you about how acceptable you think it is for people who are HIV-positive in this community to visit the HIV clinic.

Probes: To what extent do you think people in this community feel free to go to the HIV clinic in this community to receive HIV care and treatment? And why is that? To what extent do you think people from this community travel elsewhere to receive HIV care and treatment? And

why is that? How acceptable do you think it is for people in this community to start antiretroviral therapy (ART)? To what extent do you think people are able to be open about taking ART medications? What problems do you think people in this community who are HIV-positive have with starting ART? What do you think would help people to be able to visit the HIV clinic and start ART?

<u>If participant responds that he or she has previously tested for HIV:</u>

Please tell me about your HIV testing experience.

Probes: When did you test? Why did you test? Where did you test? How did you feel about your testing experience? Did you talk to anyone about your decision to test before you got tested? Have you told others that you have tested for HIV? If yes, whom have you told? If not, why not? How did people react when you told them you tested?

Can you please tell me about your experiences sharing your HIV test results with other people after you were tested?

Probes: Have you shared your HIV test results with anyone? If yes, whom? If yes, tell me about this experience – tell me about your decision to share your test results; tell me about their reaction when you shared your test results. Is there anyone you want to know your results that you have not told yet? If you did not share your results with anyone, why not? How has the availability of drugs called antiretrovirals to treat HIV affected your decision about sharing test results with people? Is there anyone whom you do not want to know your HIV test results? Today, how do you feel most people in this community react when someone close to them discloses that they are HIV-positive? To what extent do you think this has changed over time? How has this changed?

<u>If participant discloses that he/she is HIV seropositive:</u>

Probes: Can you please tell me, have you ever disclosed your HIV-positive status to anyone else? If yes, who was the first person you disclosed to? Why did you choose that person? Were there others you wanted to disclose your status to, but didn't feel you could? Can you tell me more about that? If no, can you tell me more about why you haven't felt able to disclose your status to anyone?

Have you ever received any care for HIV disease?

<u>If the participant responds that he or she has NOT previously received care for HIV:</u>

Can you tell me the main reasons why you have not yet received HIV care?

Probes: Are there any other problems or issues that have kept you from enrolling in care? Please tell me, what would make it easier for you to enroll in HIV care and treatment?

<u>If the participant responds that he or she has previously received care for HIV:</u>

Are you currently receiving regular care?

Probes: How has receiving HIV care affected your life? Where do you go for medical care? Probe for all locations. Have you ever had to change where you access HIV care at any point since you tested positive? If yes, probe for main reasons why. If not currently enrolled in care, can you tell me the main reasons you're not receiving care currently? Probe for ALL reasons, including mobility, negative provider experiences, switched to traditional medicine, etc. Please tell me, what would make it easier for you to enroll again in HIV care and treatment?

Have you ever missed HIV care appointments, or dropped out of care for a time?

Probes: What were the circumstances that led this to happen? For about how long was your care interrupted? What are the main barriers you face, to being able to make appointments? Probe for ALL reasons for missing appts.: negative experiences with providers, issues of stigma and disclosure, problems with money or transportation, etc. Are there any other reasons? Please tell me, what would make it easier for you to make appointments?

What are the main barriers you face to being able to adhere to taking your HIV medications?

Probes: Probe for ALL reasons for missing appts.: negative experiences with providers, issues of stigma and disclosure, problems with money or transportation, etc. Are there any other reasons? Please tell me, what would make it easier for you to adhere to taking your HIV medications?

<u>If the participant responds that he or she has NOT previously tested for HIV:</u>

You mentioned to me that you have never been tested for HIV before. Can you tell me more about this.

Probes: How do you feel about testing for HIV? What are some reasons you have not been tested for HIV before? Do you think you would ever want to be tested? If testing were available in your community, would you be interested in taking this test? Is there anything you think might change your mind about testing?

SEARCH Trial In-depth Interview Guide - Provider Qualitative Cohort Baseline Interview

Thank you for doing this interview. We are here to discuss your experience with providing care to patients with HIV. We want to understand your experiences and your point of view, and are not here to criticize, so please feel free to share your real experiences with us.

- What challenges do staff face in implementing streamlined ART delivery? What are the main challenges that providers perceive with regard to starting ART in patients with higher CD4 levels than national guidelines? Do these challenges differ by gender? How do the providers experience caring for patients who are sick when they start vs. mostly now healthy?
- What are providers' perceptions of the effectiveness of enhanced services?
- What do providers perceive to be the main barriers to clients receiving services?
- What do providers see as the challenges in care in that patients present and are they medical, psychosocial, or logistical? And have these barriers changed over time?

I. Experiences providing clinical care

We want to learn a little bit about your work experiences providing care to HIV/AIDS patients.

- Why were you motivated to work in providing care and counseling for HIV infected patients?
- What formal training did you receive?
- How long have you worked at this particular clinic?

II. Work setting

Please tell us about the activities that make up your day to day routine work providing counseling, care and treatment to HIV-infected patients.

- About how many patients do you counsel about ART initiation or adherence each week?
- Do you provide counseling to individuals, couples or groups?
- Do patients tend to come alone, or with family members? Does this vary by gender and age?
- What aspects of your work do you find most frustrating or difficult? What aspects are the most rewarding?

III. General ART initiation counseling content

We would like you to tell us about your conversations with patients who were eligible for antiretroviral therapy either immediately before ART initiation or during the first weeks and months of ART to understand how you provide support and care.

- Which types of patients truly merit antiretroviral therapy, in your opinion?
- When discussing ART initiation with patients who are eligible for ART but not on ART already, what are the topics are the most important and most frequently discussed?
- Have these topics changed over time?
- What special issues do women face when discussing ART initiation? What about men?

<u>For providers in intervention communities:</u>

- What are the main challenges you have faced, so far, with implementing streamlined ART delivery? Are there certain types of patients that you've especially had difficulty enrolling for ART? (For instance, has your experience varied by health status, gender, age, or other characteristic of the patients?)
- What has been your experience so far with starting ART in patients with higher CD4 levels than the national guidelines? What has it been like to care for patients who are healthy when they start ART, versus sick when they start?

IV. a. Patient-based factors in ART initiation and maintenance

Now I'd like to ask you some questions about your experiences with patients, and the problems they have initiating ART and staying engaged in HIV care and treatment.

- First of all, what do most patients know about ART, before they initiate ART? What are the main misconceptions they have? How has this changed over time?
- What do you think most patients believe to be the benefits or drawbacks of ART? How has this changed over time?
- You mentioned that [recap what provider said about patients' most common misconceptions or areas where they lack information]. How do you respond when patients have this misconception or need information? Can you please give me an example from among patients you have seen recently?
- Please tell me your opinion about the main differences between male and female patients in their knowledge, beliefs and attitudes about ART. Which are morely likely among men? Among women? What differences do you see among people in different age groups?
- What do you think people need to know about ART, in order to decide to start treatment? What other conditions need to be in place for people, in order for them to be able to initiate ART?
- What do you think most patients need to have or to believe in order to stay enrolled in care and treatment? And what do they need to have and to know in order to adhere to medications?

IV. b. Clinic-based factors in ART initiation and maintenance

We would like to learn more about the kinds of barriers that might exist at the clinic to ART initiation and engagement in care and treatment.

- From your point of view, what are the most important things that you see at the clinic, that might make it hard for patients to initiate ART? Please tell me about most people's experience with waiting times. What are the interactions like with the staff who work here? How clearly are signs laid out, showing patients at the clinic where they should go?
- What do you think the clinic can do to make ART initiation easier for patients?
- What about staying engaged in care and treatment, specifically—what could the clinic do to help patients attend their regular appointments and adhere to medications?

IV. Structural factors in ART initiation

- Patients often have difficulty starting ART, because of problems due to lack of money, transportation, work or child care – What do you think are the biggest problems for patients in this clinic?
- Can you tell us if you have any suggestions or advice for patients who face problems with transportation, money or hunger?

V. Wrap-up

- Can you tell us about perhaps the best example of a difficult case you've had, with a patient was having problems starting ART, that that you were able to address? What did you provide to that patient (knowledge, advice or other support)?
- Can you tell us about one particularly difficult instance where the patient was unable to initiate ART despite having a clinical reason to start?
- Overall, many patients also get information about HIV care and treatment from community members as well as professional doctors and counselors. Is the information that your patients receive from elsewhere in the community sometimes different from the information provided at the clinic? If so, how does it differ? What or who are the other sources of information that community members turn to? In your opinion, do most people in the community trust the information they receive from the clinic, or from others in the community? How has this changed over time?
- What additional suggestions do you have for how providers can better help HIV-infected patients?

SEARCH Trial Qualitative Cohort- SECOND INTERVIEW

[Note to team: prior to interview, prepare Summary Sheets with individualized follow-up questions. Insert the individualized follow up questions into the interview guide below, as needed. Note that questions of clarification for the informant should be asked at the beginning.]

Thank you again for taking the time to meet with me. Today, I would like to follow up with you on some of the questions we talked about 2 years ago, and then ask you some new questions. *[It would be useful here to remind the informant what you talked about in the last interview. This interview should build on what was described in this last interview. If there are points that need clarification, then those should also be asked in the beginning.]*

COMMUNITY CONTEXT

- In the last interview we had you mentioned the following major problems faced by members of this community *[Recap what the participant told you in the baseline interview here]*. Do you still feel these are major problems faced by people in this community?
- Are there any other problems that you feel people now face?
- Have there been any changes since we last spoke, in how people cope with these problems?
- [If the participants do not mention HIV/AIDS as one of the major problems, probe specifically about this.] Is HIV/AIDS a problem in this community?
  - *[If yes:]* How are people in this community coping with the problem of HIV?

COMMUNITY PERCEPTIONS / DISCUSSIONS ABOUT HIV/AIDS

- In the last interview we had you told me that people feel the following way about HIV in this community *[Recap what the participant told you in the baseline interview here]*. Do you think people feel the same way today about HIV/AIDS in this community?
- How has the way people feel about HIV/AIDS in this community changed during the past year?
  - Why do you think these changes have occurred?
- Since we last spoke have you known any other people in your community who are infected with HIV?
  - How do you feel about these people?
  - How are these people treated by others in this community?
- Have there been any changes in the way people with HIV are treated by others in this community?
- How has the availability of drugs called antiretrovirals [*or ARVS; also use language informant has previously used, e.g. 'the big medicine'*] for HIV affected this?
- If someone you know told you that they were infected with HIV, how would you react to them?
- Can you tell me about any experiences you've had with people who have disclosed their HIV status to you, since we last spoke?
  - How did you feel at the time?
  - How did you react?
- Have you talked to anyone about HIV/AIDS in your community lately?
  - If yes, can you tell me about one conversation about HIV/AIDS you have had with someone else in this community in the last 6 months. What specifically did you talk

about?
  - Why were you talking about this at that time?
- Since the time we last spoke, have you noticed any changes in your community related to attitudes and fears about HIV?
  - What changes have you noticed?
  - How did you notice these changes?
  - Where did you notice these changes?
  - Why do you think these changes occurred?

- Since we last spoke have you noticed any more discussion in your community related to HIV, testing, and treatment?
  - Since the time we last spoke, has your attitude or have your fears about HIV changed?
  - Why do you think these changes occurred?

COMMUNITY PERCEPTIONS / DISCUSSIONS ABOUT HIV TESTING & ARV THERAPY

- When we last spoke you said [*recap what informant said about whether s/he knew people taking ARVs; rephrase follow up question accordingly*]. And now, are you aware of anyone in this community who is taking drugs called antiretrovirals to treat HIV?
  - How did you learn they were taking these drugs?
  - Where do they receive their drugs?
- Do you have any concerns about these drugs that are used to treat HIV? If yes, what are your concerns?
- Since we last spoke have you noticed any more discussion in your community related to antiretroviral therapy for HIV (ARVs)?
- When we last spoke you said [*recap what informant said about whether s/he knew people who had tested for HIV; rephrase follow up question accordingly*]. And now, since we last spoke, have you known anyone in your community who has tested for HIV?
  - *If more people s/he knows have tested for HIV*: What changes have you noticed about how people feel about getting tested for HIV?
  - Why do you think these changes have occurred?
- Where did they test?
- How did you know that they tested?
- Did they tell you the results of their HIV test?
- How did you feel when they told you their test results?
- How did this person feel about their experience with testing?
- *If testing occurred within the community CHC:* Please tell me more about how community members felt about HIV testing at the CHC. How did being able to get tested at the CHC affect this community?
- Do you think that the availability of drugs called antiretrovirals to treat HIV might influence whether a person is willing to test for HIV? *Probe for reasons why / why not.*

PERSONAL EXPERIENCES WITH HIV TESTING, USE OF OTHER SERVICES & DISCLOSURE
- When we last spoke you said [*recap what informant said about his/her previous experience*

with HIV testing; rephrase follow up question accordingly]. Have you tested for HIV?

- *If yes*: when did you last test for HIV?
  - Can you tell me more about your testing experience?
  - Why did you decide to test?
  - Did you talk to anyone about your decision to test before you got tested?
  - Where did you test?
    - *If testing occurred at a CHC*: Please tell me more about your experience getting tested at the CHC. How did you feel at the time?
      - How do you think other members of this community felt about getting tested for HIV at the CHC?
      - How did being able to get tested at the CHC affect this community? Please tell me more about that.
    - *If testing occurred at home*: How did it happen that you were tested at home? Please tell me about that.
      - Please tell me about the reasons you decided to get tested at home.
      - [Clarify whether or not home-based testing was provided by SEARCH]

- How did you feel about your testing experience?
  - Did you feel that you got the support you needed during your counseling sessions?
  - Did the counselor answer all of the questions?
  - What would you have changed about the testing experience if you could change one thing?

- How did you feel about learning your HIV test results at that time?

- After you were tested for HIV did you use any specific medical/health services?
  - Tell me about the services you used.
  - Where did you use these services?
  - How did you learn about these services?
  - How did you feel about these services?
  - Are there any services you felt like you needed after you were tested, but you could not access?

- Can you tell me about your experiences sharing your HIV test results with other people after you were last tested.
  - After you were tested, did you talk to anyone about your experience with testing?
  - Have you shared your HIV test results with anyone?
    - *If yes:* whom?
      - Please tell me about your decision to share your test results: Why did you choose this person to disclose to?
      - Please tell me about their reaction when you shared your test results.
      - Were there other people you wanted to disclose your status to, but felt you couldn't? Please tell me more about that.

    - How has the availability of drugs called antiretrovirals affected your decision about sharing test results with people?
    - *If no*: What makes it difficult for you to share your results?
  - Is there anyone whom you do not want to know your HIV test results?
  - Can you tell me why you do not wish to disclose your test results to this person?
  - What makes it difficult for you to share your results with this person?
  - Did you share your HIV test results with your sexual partner?
    - [*If they mentioned that they had more than one sexual partner last time*]: Did you share your HIV test results with your other sexual partners?
    - Tell me about how you decided which partners to share your results with.
  - How did these partner(s) react when you shared your results with them?
  - How did it feel to discuss testing for HIV with your partner(s)?
  - Can you tell me why you do not wish to disclose your test results to your partner(s) (*or to a particular partner if more than one*)?
    - What makes it difficult for you to share your results with this/these partner(s)?

- *IF THE PARTICIPANT RESPONDS THAT HE OR SHE TESTED FOR HIV PRIOR TO THE YEAR 1 INTERVIEW, AND HAS NOT RE-TESTED SINCE THAT TIME*: You mentioned that you tested for HIV once before the first interview we did together, but you have not been tested again since that time. Can you tell me more about your decision not to test again for HIV?
  - Do you think there is any reason for a person to test more than one time for HIV?
  - If yes, why have you decided not to test again for HIV?
  - Do you think that you may decide to test again at some point? What would be the reasons that would make it likely for you to get tested again?

<u>PERSONAL EXPERIENCES WITH HIV CARE AND TREATMENT [FOR HIV+ INDIVIDUALS]</u>

- Have you ever received any care for HIV?
- *If the participant responds that he or she has NOT previously received care for HIV*: Can you tell me the main reasons why you have not yet received HIV care?
  - Are there any other problems or issues that have kept you from enrolling in care?
  - Please tell me, what would make it easier for you to enroll in HIV care and treatment?

[FOR PARTICIPANTS WHO HAVE NEVER ENROLLED IN HIV CARE & TREATMENT, SKIP TO NEXT SECTION]

- *If the participant responds that he or she has previously received care for HIV*: Are you currently receiving regular care?
  - How has receiving HIV care affected your life?
  - Can you tell me please about the drug treatment you are receiving for HIV/AIDS? *Probe whether informant knows what ARV meds are, whether they know what meds they are taking.*
    - *If receiving ARV therapy*: Please tell me about how you learned you were eligible for

ARV therapy. Where and when did you learn you were eligible to begin taking ARVs?
- Who spoke to you about your eligibility for ARV therapy*? Probe for cadre of health care provider and health centre.*
- How has taking ARVs affected your life?

▪ *If not on ARV therapy*: Can you tell me what you have been told about your eligibility or ineligibility for ARV therapy?
- *If previously told s/he is eligible for ARV therapy, but has not yet started*: Can you please tell about your reasons for not starting ARV therapy?
- Are there any other reasons you have not started taking ARVs? *Probe for ALL reasons*.

  - Where do you go for medical care? *Probe for all locations*.
  - Have you ever had to change where you access HIV care at any point since you tested positive? *If yes, probe for main reasons why*.

- *If participant not currently enrolled in care:* Can you tell me the main reasons you're not receiving care currently? *Probe for ALL reasons*.
  - Please tell me, what would make it easier for you to enroll again in HIV care and treatment?

- Can you please tell me about your experience learning about your viral load?
  - When and where did you first learn about viral load, and what it means?
  - How has it affected you, to know about your viral load?
  - How important is it to you, to know your viral load?
- Have you ever missed HIV care appointments, or dropped out of care for a time? Please tell me about that.
  - What were the circumstances that led this to happen?
  - For about how long was your care interrupted?
  - What are the main barriers you face, to being able to make appointments? *Probe for ALL reasons*.
  - Are there any other reasons?
  - Please tell me, what would make it easier for you to make appointments?
- What are the main barriers you face to being able to adhere to taking your HIV medications? *Probe for ALL reasons*.
  - Are there any other reasons?
  - Please tell me, what would make it easier for you to adhere to taking your HIV medications?

<u>RELATIONSHIPS & DISCUSSIONS ABOUT HIV WITH PARTNERS [ALL PARTICIPANTS]</u>

- In the last interview we had together, you told me that you had _______ sexual partner(s) [*Recap what participant mentioned about their sexual partnerships, without referring to a*

number of partners]. Are you with the same partner(s) that you were with before?
- Have you had any new sexual partners since we last talked?
- If you are no longer with your former partner(s), can you tell me why your relationships ended?

[*For the following questions, first refer to main partner, then repeat questions for other current/ recent partners*]
- In the last 6 months have you and your partner ever talked about HIV and your risk of HIV?
    - o *If yes*: Can you tell me about the conversations you have had with your partner about HIV?
    - o *If informant has not talked to partner(s) about HIV or risk of HIV in the last year*: Can you tell me why it is difficult to talk to your partner about HIV?

- *For HIV-negative participants:* Do you feel that you are at risk for HIV because of this partner?
    - o *If yes*: Tell me why you think you may be at risk.
        - ▪ Do you do anything in your relationship to try to minimize this risk?

- *For HIV-positive participants:* Do you feel any concern about re-infection with a different strain of HIV?
    - o *If yes*: Tell me why you think you may be at risk.
        - ▪ Do you do anything in your relationship to try to minimize this risk?
- Please tell me about any concerns you feel about infecting your partner with HIV?
        - ▪ Do you do anything in your relationship to try to minimize this risk?

- *ALL participants*: How has the availability of drugs called antiretrovirals to treat HIV affected this?
- Is there anything else you would like to do in your relationship to minimize your risk, yet you feel you are unable to do?
    - o *If yes*: Can you tell me why you feel like you are unable to do this with your partner(s).

- Have you talked to your partner about getting tested for HIV in the last year?
    - o *If yes*: Can you tell me what you and your partner discussed.
        - ▪ How did it feel to discuss testing with your partner?
        - ▪ How does your partner feel about getting HIV tested?
    - o *If no*: Can you tell me why it is difficult to discuss HIV testing with your partner?

- *For HIV-positive participants*: Have you talked to your partner about accessing HIV care and treatment in the last year?
    - o *If yes*: Can you tell me what you and your partner discussed.
    - o *If no*: Can you tell me why it is difficult to discuss HIV care and treatment with your partner?

Thank you for sharing your time with me. The information you have given will be very useful for the study.

SEARCH Trial In-depth Interview Guide – Community Leader Cohort Follow-up

[Note prior to interview, ethnographer prepares a summary sheet with main findings from baseline interview. This is used to prepare individualized follow-up questions as needed.]

Thank you again for taking the time to meet with me. Today, I would like to follow up with you on some of the questions we talked about last time, and then ask you some new questions. [It would be useful here for the ethnographer to remind the informant what they talked about in the last interview. This interview should build on what was described in this last interview. If there are points that need clarification, then those should also be asked in the beginning.]

In the interview we had last year, you mentioned that you undertook the following main activities to prepare the community to participate in the community health fair (recap what the participant told you in the baseline interview here). What about this year: what activities did you and others in the community undertake to get people to participate?

Probes: What do you think were the main ways that people in the community heard about the fair this year? Were there any other actions that you or others undertook to get people in the community to come to the fair?

Let's talk about the health fair this year. Please tell me what aspects of the fair went well, in your opinion? Which aspects of the fair did not go as well as you'd hoped?

Probes: In your opinion, what were the main reasons that community members participated in the health fair? For those that did not attend, what do you think were the main reasons they did not? Which groups of people in this community were more difficult to mobilize to attend the health fair, and why? How successful were you in getting these groups of people to attend the fair? How do you think they can better be motivated to participate in next year's fair?

Please tell me your thoughts about next year's health fair.

Probes: How do you think the fair should be promoted, next year? What will you repeat, and what things will you do differently to promote the fair among community members? For next year's fair, what aspects of the fair should be the same? How can the health fair be improved?

Topic 2: Attitudes, beliefs and social norms surrounding HIV, testing, disclosure, and ART

Now I would like to talk with you about health in this community, including the knowledge and beliefs people in this community have about HIV/AIDS, as well as your own experiences and opinions. If there are any questions that make you feel uncomfortable, you should feel free to tell me and we can skip over those questions.

In the last interview we had you mentioned the following major problems faced by members of this community (recap what the participant told you in the baseline interview here). Do you still feel these are major problems faced by people in this community?

Probes: Are there any other problems that you feel people now face? Have there been any changes in how people cope with these problems in the last two years? If the participants do

not mention HIV/AIDS as one of the major problems, probe specifically about this. Is HIV/AIDS a problem in this community? If yes, how are people in this community coping with the problem of HIV?

Since the time we last spoke, have you noticed any changes in your community related to attitudes and fears about HIV?

Probes: What changes have you noticed? How did you notice these changes? Where did you notice these changes? Why do you think these changes occurred? Since we last spoke have you noticed any more discussion in your community related to HIV, testing, and treatment? Since the time we last spoke, has your attitude or have your fears about HIV changed? Why do you think these changes occurred?

Since we last spoke are you aware of anyone in this community who is taking drugs called antiretrovirals to treat HIV?

Probes: How did you learn they were taking these drugs? Where do they receive their drugs? Do you have any concerns about these drugs that are used to treat HIV? If yes, what are your concerns? How does the availability of antiretrovirals affect the way people in your community react to HIV infected people? Can you tell me, what are all the benefits of antiretroviral therapy? To what extent do you think people in this community know that ART can help HIV-positive people to not transmit the virus to others?

Since we last spoke, have you known anyone in your community who has tested for HIV?

Probes: Where did they test? How did you know that they tested? Did they tell you the results of their HIV test? How did you feel when they told you their test results? How did this person feel about their experience with testing? Do you think that the availability of drugs called antiretrovirals to treat HIV might influence whether a person is willing to test for HIV?

Since we last spoke, have there been any changes in how free people feel in this community to visit the HIV clinic here to receive HIV care and treatment?

Probes: What accounts for those changes? To what extent do you think people from this community travel elsewhere to receive HIV care and treatment? And why is that? To what extent have there been any changes in how acceptable it is for people in this community to start antiretroviral therapy (ART)? What about taking ART medications: to what extent have there been any changes in how open people are able to be about that? What problems do you think people in this community who are HIV-positive continue to have with starting ART? What do you think would help people to be able to visit the HIV clinic and start ART?

In the last interview we had you told me that (recap what the participant told about their experience with HIV testing, i.e. whether they had been tested, and whether they tested HIV-positive. Use summary of previous findings to tailor questions in this section).

Probes: (If did not previously test): Have you tested for HIV? (If previously tested for HIV): When did you last test for HIV?

<u>If the participant responds that he or she has tested for HIV in the past year:</u>

Can you tell me more about your testing experience?

Probes: Why did you decide to test? Did you talk to anyone about your decision to test before you got tested? Where did you test? How did you feel about your testing experience? Did you feel that you got the support you needed during your counseling sessions? Did the counselor answer all of the questions? What would you have changed about the testing experience if you could change one thing? How did you feel learning your HIV test results at that time? Have you told others that you have tested for HIV? If yes, whom have you told? If not, why not? How did people react when you told them you tested?

Today, how do you feel most people in this community react when someone close to them discloses that they are HIV-positive?

Probes: To what extent do you think this has changed over time? How has this changed?

After you were tested for HIV did you use any specific medical/health services?

Probes: Tell me about the services you used. Where did you use these services? How did you learn about these services? How did you feel about these services? Are there any services you felt like you needed after you were tested, but you could not access?

Can you please tell me about your experiences sharing your HIV test results with other people after you were tested?

Probes: Have you shared your HIV test results with anyone? If yes, whom? If yes, tell me about this experience – tell me about your decision to share your test results; tell me about their reaction when you shared your test results. Is there anyone you want to know your results that you have not told yet? If you did not share your results with anyone, why not? How has the availability of drugs called antiretrovirals to treat HIV affected your decision about sharing test results with people? Is there anyone whom you do not want to know your HIV test results?

<u>If participant disclosed in this or previous interview that he/she is HIV seropositive:</u>

Probes: (If in previous interview, participant had not ever disclosed status): Can you please tell me, have you disclosed your HIV-positive status to anyone else? If yes, who was the first person you disclosed to? Why did you choose that person? Were there others you wanted to disclose your status to, but didn't feel you could? Can you tell me more about that? (If no): Can you tell me more about why you haven't felt able to disclose your status to anyone?

Have you ever received any care for HIV disease?

<u>If the participant responds that he or she has NOT previously received care for HIV:</u>

Can you tell me the main reasons why you have not yet received HIV care?

Probes: Are there any other problems or issues that have kept you from enrolling in care? Please tell me, what would make it easier for you to enroll in HIV care and treatment?

<u>If the participant responds that he or she has previously received care for HIV:</u>

Are you currently receiving regular care?

Probes: How has receiving HIV care affected your life? Where do you go for medical care? Probe for all locations. Have you ever had to change where you access HIV care at any point since you tested positive? If yes, probe for main reasons why. If not currently enrolled in care, can you tell me the main reasons you're not receiving care currently? Probe for ALL reasons, including mobility, negative provider experiences, switched to traditional medicine, etc. Please tell me, what would make it easier for you to enroll again in HIV care and treatment?

Have you ever missed HIV care appointments, or dropped out of care for a time?
Probes: What were the circumstances that led this to happen? For about how long was your care interrupted? What are the main barriers you face, to being able to make appointments? Probe for ALL reasons for missing appts.: negative experiences with providers, issues of stigma and disclosure, problems with money or transportation, etc. Are there any other reasons? Please tell me, what would make it easier for you to make appointments?

What are the main barriers you face to being able to adhere to taking your HIV medications?
Probes: Probe for ALL reasons for missing appts.: negative experiences with providers, issues of stigma and disclosure, problems with money or transportation, etc. Are there any other reasons? Please tell me, what would make it easier for you to adhere to taking your HIV medications?

<u>If the participant responds that he or she tested for HIV prior to baseline, but has not re-tested since that time:</u>

You mentioned that you tested for HIV once before the first interview we did together, but you have not been tested again since that time. Can you tell me more about your decision not to test again for HIV?
Probes: Do you think there is any reason for a person to test more than one time for HIV? If yes, why have you decided not to test again for HIV? Do you think that you may decide to test again at some point? If testing were available in your community, do you think you would test again for HIV?

<u>If the participant responds that he or she has not tested for HIV:</u>

You mentioned to me that you have never been tested for HIV before. Can you tell me more about this.
Probes: How do you feel about taking an HIV test? What are some reasons you have not been tested for HIV before? Do you think you would ever want to be tested? If testing were available in your community, would you be interested in taking this test? Is there anything you think might change your mind about testing?

SEARCH Trial In-depth Interview Guide - Provider Qualitative Cohort Follow-up Interview

[*Note prior to interview, ethnographer prepares a summary sheet with main findings from previous interview. This is used to prepare individualized follow-up questions. These individualized follow up questions will be inserted into the interview guide below, as needed*.] Thank you for doing this interview. We are here to discuss your experience with providing care to patients with HIV. We want to understand your experiences and your point of view, and are not here to criticize, so please feel free to share your real experiences with us.

I. Experiences providing clinical care

We want to learn a little bit about your work experiences providing care to HIV/AIDS patients. [*Recap what the provider previously said about their training and how long they have worked in the clinic. Probe for information on any additional training or change in position/cadre*.]

- How long have you worked at this particular clinic?
- Please tell us about the activities that make up your day to day routine work providing counseling, care and treatment to HIV-infected patients, at the current time. About how many patients do you counsel about ART initiation or adherence each week?
- Do you provide counseling to individuals, couples or groups?
- Do patients tend to come alone, or with family members? Does this vary by gender and age? [*If so*: Can you tell me more about that?]
- What do you feel are the most rewarding and fulfilling aspects of your job?
- What are the things that make it hard for you to do your best work, or that decrease your motivation and morale?

II. General ART initiation counseling content

We would like you to tell us about your conversations with patients who were eligible for antiretroviral therapy either immediately before ART initiation or during the first weeks and months of ART to understand how you provide support and care.

- Which types of patients truly merit antiretroviral therapy, in your opinion?
- When discussing ART initiation with patients who are eligible for ART but not on ART already, what are the topics are the most important and most frequently discussed?
- How have these topics changed over time?
- What special issues do women face when discussing ART initiation? What about men?

III. Viral load counseling

Note: Ask these questions for all intervention community providers. For control community providers, first ask about whether VL testing and counseling happens at the health center, and for which patients. If viral load testing and counseling happens at site, proceed (otherwise, skip to section IV).

- What has been the patients' experience regarding learning about their viral loads?

- Can you tell me please, how do you explain to patients what viral load testing means? Please give me an example of how you typically describe HIV viral load to patients.
    - What kinds of questions do patients have about viral load testing?
- How has the patient knowing their viral load affected them?
- How has knowing the patient's viral load level affected your care of the patient?

IV. a. Patient-based factors in ART initiation and maintenance

Now I'd like to ask you some questions about your experiences with patients, and the barriers they face with initiating ART and staying engaged in HIV care and treatment, at the present time. [*Recap from interview summary main points of what provider said in previous interview*.]

- First of all, what do most patients know about ART, before they initiate ART?
    - What are the main misconceptions they have?
    - How has this changed since we last spoke, if it has changed?
- What do you think most patients believe to be the benefits or drawbacks of ART?
    - How has this changed since we last spoke, if it has changed?
- You mentioned that [*recap what provider said about patients' most common misconceptions or areas where they lack information*].
    - How do you respond when patients have this misconception or need information?
    - Can you please give me an example from among patients you have seen recently?
- Please tell me your opinion about the main differences between male and female patients in their knowledge, beliefs and attitudes about ART.
    - Which are more common among men?
    - What about among women?
    - What differences do you see among people in different age groups?
- What do you think people need to know about ART, in order to decide to start treatment?
    - What other conditions need to be in place for people, in order for them to be able to initiate ART?
- What do you think most patients need to have or to believe in order to stay enrolled in care and treatment?
    - *Probe for the main challenges in care that patients present.*
    - *Probe for whether they are mostly medical, psychosocial, or logistical*
    - *How have these barriers changed since we last spoke, if they have changed?*
    - *Please tell me about more about what other things help patient to stay engaged in care?*
- And what do they need to have and to know in order to adhere to medications?
    - *Probe for the main challenges in care that patients present.*
    - *Probe for whether they are mostly medical, psychosocial, or logistical*
    - *How have these barriers changed since we last spoke, if they have changed?*
    - *Please tell me about more about what other things help patient to stay engaged in care?*

<u>For providers in intervention communities:</u>

- What are the main challenges you have faced, so far, with implementing streamlined ART delivery?
    - Are there certain types of patients that you've especially had difficulty enrolling for ART? (For instance, has your experience varied by health status, gender, age, or other characteristic of the patients?)
- What has been your experience so far with starting ART in patients with higher CD4 levels than the national guidelines?
    - What has it been like to care for patients who are healthy when they start ART, versus sick when they start?

- What aspects of the early ART intervention have been more difficult to implement? *Probe as needed: please tell me more about that.*
    - Which have been easier?

IV. b. Clinic-based factors in ART initiation and maintenance

We would like to learn more about the kinds of barriers that might exist *at the clinic* to ART initiation and engagement in care and treatment, at the present time. (Recap from interview summary main points of what provider said in previous interview.)

- From your point of view, what are the most important things that you see at the clinic, that might make it hard for patients to initiate ART?
    - Please tell me about most people's experience with waiting times.
    - What are the interactions like with the staff who work here?
    - How clearly are signs laid out, showing patients at the clinic where they should go?
- What do you think the clinic can do to make ART initiation easier for patients?
- What about staying engaged in care and treatment, specifically—what could the clinic do to help patients attend their regular appointments and adhere to medications?
- Since we last spoke, how have clinic operations changed for the better or for the worse, with respect to quality of care for HIV-infected patients?

IV. c. Structural factors in ART initiation

- Patients often have difficulty starting ART, because of problems due to lack of money, transportation, work or child care – What do you think are the biggest problems for patients in this clinic?
- Can you tell us if you have any suggestions or advice for patients who face problems with transportation, money or hunger?

V. Wrap-up

- Can you tell us about perhaps the best example of a difficult case you've had, with a patient was having problems starting ART, that that you were able to address?
    - What did you provide to that patient (knowledge, advice or other support)?
- Can you tell us about one particularly difficult instance where the patient was unable to initiate ART despite having a clinical reason to start?

- Overall, many patients also get information about HIV care and treatment from community members as well as professional doctors and counselors. Is the information that your patients receive from elsewhere in the community sometimes different from the information provided at the clinic? If so, how does it differ?
    - What or who are the other sources of information that community members turn to?
    - In your opinion, do most people in the community trust the information they receive from the clinic, or from others in the community?
    - How has this changed over time?
- What additional suggestions do you have for how providers can better help HIV-infected patients?
